# Successful treatment of a stage IIIC small-cell carcinoma of the ovary hypercalcemic subtype using multi-modality therapeutic approach

**DOI:** 10.3332/ecancer.2018.832

**Published:** 2018-05-03

**Authors:** Qian Qin, Veronica B Ajewole, Tiffany G Sheu, Rachel Donohue, Monisha Singh

**Affiliations:** 1 Department of Internal Medicine, Houston Methodist Hospital, Houston, TX 77030, USA; 2 Houston Methodist Cancer Centre, Houston Methodist Hospital, Houston, TX 77030, USA; 3 Department of Pathology and Genomic Medicine, Houston Methodist Hospital, Houston, TX 77030, USA; 4 Department of Pharmacy Practice, Texas Southern University College of Pharmacy and Health Sciences, Houston, TX 77004, USA

**Keywords:** small cell carcinoma of the ovary hypercalcemic subtype, SCCOHT, advance stage, therapy

## Abstract

Small-cell carcinoma of the ovary, hypercalcemic type (SCCOHT) is a rare but highly undifferentiated, aggressive malignancy that primarily affects young women. Due to its early onset, unclear familial history and vague presenting symptoms, most SCCOHT patients present late with advanced disease. The prognosis is extremely poor, with <10% disease-free survival for advanced stages. Although several therapeutic regimens have been proposed, to date there is no consensus on the optimal strategy.

Here, we describe a successful case of advanced-stage SCCOHT of the left ovary treated with cytoreductive surgery, semi-intense chemotherapy, high-dose consolidative chemotherapy, autologous hematopoietic stem cell transplantation and pelvic radiation with long-term survival. Given the almost universal mortality of advanced SCCOHT in long-term follow-up, we believe this case highlights the importance of prompt diagnosis when a young patient presents with abdominal swelling and hypercalcemia as well as early, aggressive, combined modality treatment. This case is also especially remarkable given the patient underwent fertility preservation surgery, which is not recommended by most of the current literature. However, as therapies improve and more young patients may survive SCCOHT, the question of fertility will increase in relevance. We believe the pros and cons of conservation should be discussed in detail with the patient.

## Introduction

First described by Dickersin *et al* [1] in 1982, small-cell carcinoma of the ovary, hypercalcemic type (SCCOHT) is a rare but highly undifferentiated, aggressive malignancy [1]. Although the age at diagnosis ranges from 14 months to 71 years, it primarily affects young women with a median of 23 years [1–4]. Approximately ~60% of SCCOHT cases present with paraneoplastic hypercalcemia [2]. Due to its early onset, unclear familial history and vague presenting symptoms, most SCCOHT patients present late with advanced disease [2–4]. The prognosis is generally very poor, with a 33% disease-free survival for stage IA disease and exponentially poorer prognosis (<10%) in more advanced stages [2, 3]. Diagnosis is difficult owing to the unclear cell lineage that SCCOHT arises from and its histological similarity to a wide variety of tumours [2–6]. The disease’s tendency for rapid progression and high recurrence also makes treatment a challenge. Several therapeutic regimens have been proposed; however, there is no consensus on the optimal strategy [3, 7–10].

We describe a previously healthy 19-year-old female who was diagnosed with stage IIIC SCCOHT. She successfully underwent semi-intensive chemotherapy treatment, conditioning chemotherapy, autologous hematopoietic stem cell transplantation (HSCT), as well as pelvic/para-aortic radiation based on the novel regimen designed by Pautier *et al* [7] the only prospective trial to date treating 27 patients with SCCOHT [7]. Our patient remains disease free 8 years from initial diagnosis.

## Case presentation

**Figure 1.**
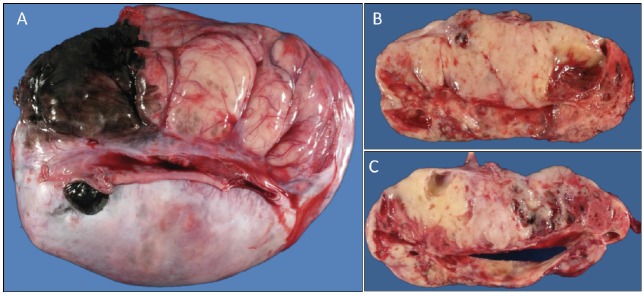
(a): Gross image of the external surface of the left ovary (20.5 cm, 1.6 kg). (b and c): representative cut surfaces of the left ovary.

**Figure 2.**
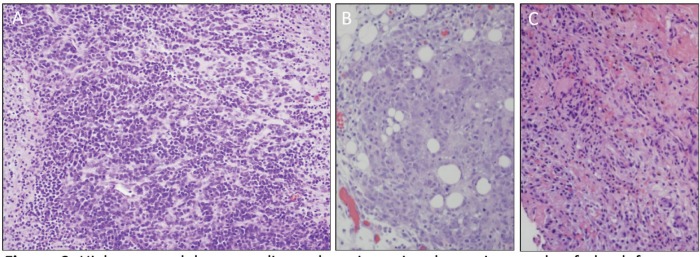
High-powered hematoxylin and eosin stain photomicrograph of the left ovary, omentum, and posterior cul de sac peritoneum (a, b and c, respectively) all showing diffuse sheet-like architecture of small round cells with scant cytoplasm, hyperchromatic nuclei and small nucleoli.

**Figure 3.**
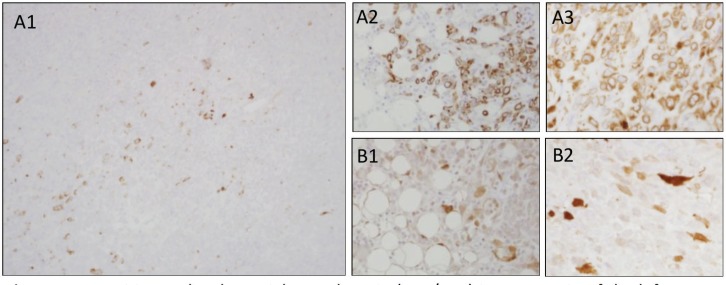
(a): Positive molecular weight cytokeratin (AE1/AE3) immunostain of the left ovary, omentum and posterior cul de sac peritoneum (a1, a2 and a3, respectively). (b): Positive calretinin immunostaining of the omentum and posterior cul de sac peritoneum (b1 and b2, respectively).

A previously healthy 19-year-old G0P0 female initially presented with several months history of increasing abdominal distension, weight loss, constipation and generalised malaise. Computerised tomography (CT) scan revealed a 20 cm mass extending from the left ovary. The patient was also tachycardic to the 120 s with a fever of 101.2 ^o^F and mild hypotension. The patient had a negative pregnancy test, normal alpha fetoprotein level at 2.3 ng/mL, normal carcinoembryonic antigen at <0.5 ng/mL but an elevated cancer antigen (CA) 125 at 375 U/mL (normal <30 U/mL) raising concerns for gynaecological malignancy. The patient also had hypercalcemia with uncorrected serum calcium of 16 mg/dL, normal thyroid stimulating hormone, a low parathyroid hormone (PTH), but an elevated PTH related peptide level at 5.0 pmol/L. She underwent left salpingo-oophorectomy, omentectomy, bilateral pelvic lymph node dissection, bilateral para-aortic lymph node dissection and peritoneal biopsy. The right ovary and uterus were spared in an attempt to preserve endocrine and fertility functions. The resected tumour size was 20.5 cm, weight was 1602 g, with large areas of necrosis but negative resection margins and uninvolved left fallopian tube (Figure 1). One of the fourteen dissected lymph nodes was positive. Pathology slide revealed SCCOHT and the patient was diagnosed with stage IIIC disease (Figures 2 and 3). The patient reported no other personal or family histories of malignancies.

Given the aggressive nature of the disease and a lack of therapeutic consensus, the patient was initiated on a regimen based on that reported by Pautier *et al* [7] after correspondences with the author and tumour board discussion. She received six cycles of semi-intensive chemotherapy with cisplatin 100 mg/m^2^ and adriamycin 40 mg/m^2^ on day one, as well as etoposide 75 mg/m^2^ and cyclophosphamide 300 mg/m^2^ on days 1–3. The regimen was given for every 21 days with pegfilgrastim support. The patient experienced fatigue, nausea, vomiting and diarrhoea, but no symptoms above grade two. These were well controlled with antiemetic and antidiarrhoeal medications. A CT scan of the chest, abdomen and pelvis post chemotherapy showed no residual disease. Serum calcium, PTH related peptide and CA125 also normalised to 9.0 mg/dL, <0.2 pmol/L and 6 U/mL, respectively.

The patient was considered to have a complete response. A bone marrow biopsy showed no pathological involvement and the process for autologous HSCT was initiated. A total of 7.845 × 10^6^ CD34/kg stem cells were collected after stem cell mobilisation with filgrastim. The patient subsequently underwent high-dose conditioning chemotherapy with carboplatin 400 mg/m^2^, etoposide 450 mg/m^2^ and cyclophosphamide 1600 mg/m^2^ followed by autologous HSCT. Her transplantation course was complicated by neutropenic fever requiring broad spectrum antibiotics, as well as odynophagia and severe mucositis with poor appetite requiring short-term total parenteral nutrition. Her symptoms slowly improved with full engraftment approximately 2 weeks post transplantation. She was discharged home in a stable condition. The patient subsequently completed 30 days of radiation therapy to the pelvic and para-aortic areas via intensity modulated radiation therapy with a cumulative dose of 40 Gy without complications. Her follow-up over the subsequent years showed no disease recurrence.

## Discussion/literature review

Here, we describe a 19-year-old patient with stage IIIC SCCOHT who has remained disease free for 8 years since initial diagnosis. This is a remarkable outcome considering the unfavourable characteristics at diagnosis (tumour size >10 cm, age <30 years and elevated pre-operative calcium) and the advance disease stage. Most literature agrees upon the almost universal mortality of advanced SCCOHT in long-term follow-up [2, 3, 8, 11]. This extremely poor prognosis warrants closer evaluation of the clinical symptoms, pathological findings, treatment modalities and post-therapy surveillance of SCCOHT.

In the landmark study by Young *et al* [2], the clinical and pathological features of 150 patients with SCCOHT were analysed. The tumour is almost always unilateral with right-side predominance and a mean tumour size of 15 cm^2^. Patients usually presented with vague symptoms including subacute nausea and vomiting, abdominal pain, swelling, fatigue and lethargy similar to other ovarian malignancies. However, the median age of diagnosis for SCCO is 23 years with hypercalcemia observed in 62% of the patients [2]. These clinical findings have been documented in subsequent publications [3,11]. As such, a young patient presenting with a negative pregnancy test but abdominal swelling and hypercalcemia should raise concerns for SCCOHT.

The pathological basis of SCCOHT remains unclear but suggests a primitive germ cell origin. The initial histopathology described in 1982 reported neoplastic cells with structural similarities to those of epithelial nature. However, they lacked any of the specific identifying characteristics associated with germline, sex cord, Sertoli or granulosa cells [1]. The cells were simply described as diffuse infiltrates of small cells with scant cytoplasm and hyperchromatic nuclei, and the malignancy was deemed small-cell carcinoma of the ovary with histopathology to be determined [1]. The subsequent study by Ulbright *et al* [13] supports a germ cell origin, especially related to yolk sac tumours, based on similar epidemiological distributions as well as light microscopy, immunohistochemical, and ultrastructural examinations. A recent comprehensive literature review, whole tissue staining and tissue microarray of SCCOHT cases by McCluggage *et al* [14] further evidenced its primitive germ cell origin as many demonstrated SALL4 positivity. Recent studies also suggest germline or somatic deleterious mutation of SMARCA4 as a major propagator of SCCOHT [15–18]. Especially validating is the comprehensive genetic profiling by Lin *et al* [19] which revealed SMARCA4 inactivation in 94% of the analysed SCCOHT cases with 62.5% predicted to be germline mutations. The study also revealed very few re-occurring genetic alterations, further supporting SMARCA4 as the main driver in SCCOHT tumourgenesis [19].

Despite these advances, there is still a lack of consensus in the treatment of SCCOHT. In a retrospective study by Callegaro-Filho *et al* [11], the treatment and disease course of 47 patients with SCCOHT were analysed with varying surgical debulking, chemotherapy regimens, plus or minus radiation and autologous HSCT. Recurrent disease was unfortunately observed in 74.5% of the cases [11]. Given the correlation of SCCOHT to germ cell tumours and prominent data suggesting benefit of high-dose chemotherapy with autologous HSCT in germ cell tumours, the recent years have seen a move towards more intensive, multi-modality treatment regimens with higher successes [8, 9, 15]. Especially in Pautier *et al* [7], the only prospective study to date, the patients underwent nonconservative debulking surgery, semi-intensive chemotherapy (cisplatin, adriamycin, etoposide and cyclophosphamide), high-dose consolidation chemotherapy (carboplatin, etoposide and cyclophosphamide) followed by autologous HSCT [7]. The outcomes were better for early-stage patients although late-stage diseases remain difficult to manage [7]. In their concluding remarks, Pautier *et al* [7] recommended considering increasing doses of initial chemotherapy as well as the addition of pelvic radiation therapy to improve survival. We thus added radiation therapy to our regimen and believe this helped decrease local recurrence and improve our patient’s clinical outcome. Other currently explored modalities include targeted therapy with EZH2 inhibitors and immunotherapy with programmed death 1/programmed death-ligand 1 (PD-1/PD-L1) inhibitors [20, 21]. Specifically, the above-mentioned SMARCA4 alteration leads to BRG1 loss-of-function, which may allow for sensitivity to tazemetostat, an EZH2 inhibitor currently implemented in a phase II trial that includes SCCOHT (clinicaltrials.gov, NCT02601950) [19, 20]. PD-1 and PD-L1 inhibitors in turn may offset adaptive immune evasion of the SCCOHT tumour cells [21].

Finally, although SCCOHT is a disease with >95% unilateral ovarian involvement, most current literature argues against fertility conserving surgery [1, 7]. However, given the young population and long-lasting endocrine effects, bilateral oophorectomy should not be performed lightly. Especially as the above-mentioned therapies improve and more patients survive SCCOHT, the question of preservation will increase in relevance. Although very few cases exist on its success, our patient with stage IIIC disease underwent cytoreductive but fertility preserving surgery. The patient declined ovarian protection with gonadotropin-releasing hormone (GnRH) agonist or cryopreservation prior to chemotherapy; however, she now has the return of normal menstruation and is currently seeking fertility counselling to bear children [22, 23]. While a more definitive answer is difficult to achieve without a noninferiority trial, we believe the conversation of pros and cons regarding fertility conservation should be discussed with patients presenting with SCCOHT.

## Conclusion

We have presented a successful case of advanced-stage SCCOHT of the left ovary treated with cytoreductive surgery but with uterus and contralateral ovary preservation, intense chemotherapy, high-dose consolidative chemotherapy, autologous HSCT and pelvic radiation with long-term survival. Due to its early onset, aggressive disease course and poor prognosis, we believe this early, high intensity, multimodality treatment regimen should be considered when approaching SCCOHT.

## Conflicts of interest and funding statement

All authors have no conflict of interests and nothing to disclose. No funding was accepted for this case study.
